# The Purified Extract from the Medicinal Plant *Bacopa monnieri*, Bacopaside II, Inhibits Growth of Colon Cancer Cells In Vitro by Inducing Cell Cycle Arrest and Apoptosis

**DOI:** 10.3390/cells7070081

**Published:** 2018-07-21

**Authors:** Eric Smith, Helen M. Palethorpe, Yoko Tomita, Jinxin V. Pei, Amanda R. Townsend, Timothy J. Price, Joanne P. Young, Andrea J. Yool, Jennifer E. Hardingham

**Affiliations:** 1 Molecular Oncology, Basil Hetzel Institute, The Queen Elizabeth Hospital, Woodville South SA 5011, Australia; eric.smith@adelaide.edu.au (E.S.); helen.palethorpe@adelaide.edu.au (H.M.P.); yoko.tomita@sa.gov.au (Y.T.); 2 Adelaide Medical School, University of Adelaide, Adelaide SA 5000, Australia; jinxin.pei@adelaide.edu.au (J.V.P.); amanda.townsend@sa.gov.au (A.R.T.); timothy.price@sa.gov.au (T.J.P.); joanne.young@adelaide.edu.au (J.P.Y.); andrea.yool@adelaide.edu.au (A.J.Y.); 3 Medical Oncology, The Queen Elizabeth Hospital, Woodville South SA 5011, Australia

**Keywords:** *Bacopa monnieri*, bacopaside II, colorectal cancer, anti-tumour agents, aquaporin-1, cell cycle arrest, apoptosis

## Abstract

Aquaporin-1 (AQP1), a transmembrane pore-forming molecule, facilitates the rapid movement of water and small solutes across cell membranes. We have previously shown that bacopaside II, an extract from the medicinal herb *Bacopa monnieri*, blocks the AQP1 water channel and impairs migration of cells that express AQP1. The aim of this study was to further elucidate the anti-tumour potential of bacopaside II in colon cancer cells. Expression of AQP1 in HT-29, SW480, SW620 and HCT116 was determined by quantitative PCR and western immunoblot. Cells were treated with bacopaside II, and morphology, growth, autophagy, cell cycle and apoptosis assessed by time-lapse microscopy, crystal violet, acridine orange, propidium iodide (PI) and annexin V/PI staining respectively. AQP1 expression was significantly higher in HT-29 than SW480, SW620 and HCT116. Bacopaside II significantly reduced growth at ≥20 µM for HT-29 and ≥15 µM for SW480, SW620 and HCT116. Inhibition of HT-29 at 20 µM was primarily mediated by G0/G1 cell cycle arrest, and at 30 µM by G2/M arrest and apoptosis. Inhibition of SW480, SW620 and HCT116 at ≥15 µM was mediated by G2/M arrest and apoptosis. These results are the first to show that bacopaside II inhibits colon cancer cell growth by inducing cell cycle arrest and apoptosis.

## 1. Introduction

Worldwide, colorectal cancer (CRC) is the third most commonly diagnosed cancer and is a leading cause of cancer-related deaths [1]. At diagnosis, up to 25% of patients present with metastatic (stage IV) disease. Up to 25% of patients diagnosed with early localised (stage I or II) disease and 50% with regional spread to lymph nodes or adjacent organs (stage III) eventually relapse with overt metastatic disease following surgery with curative intent [2]. Despite recent advances in treatment, patients with metastatic disease currently have 5-year survival rates of less than 15%, and a median overall survival of approximately 30 months [3,4]. There is a need to improve treatment regimens for patients with, or at risk of developing, metastatic CRC.

*Bacopa monnieri*, commonly known as Brahmi, has been used in Ayurvedic medicine for thousands of years as a memory enhancer, sedative, analgesic, anti-epileptic and anti-inflammatory agent. The plant extract and its isolated major active principles (bacosides) have been extensively investigated for their nootropic effects and exhibit minimal observable adverse effects at standard dosages (reviewed in [5]). The known active chemical constituents are dammarane-type triterpenoid saponins with jujubogenin or pseudo-jujubogenin moieties as aglycone units [6,7]. These saponins include bacosides A1-A3, bacopasaponins A-G, and bacopasides I-XII [8,9]. The majority of studies have been conducted using plant extracts, of which the individual constituents and their concentrations may vary depending upon the extraction process.

Plant extracts of *Bacopa monnieri* have been shown to be cytotoxic both in vitro and in vivo in several cancer cell lines including those of colon, lung and breast [10,11]. However, there are only limited studies that have specifically investigated the anti-tumour effects of purified bacopaside II. We, and others, have previously reported that bacopaside II was cytotoxic in vitro for a range of cancer cell lines [12,13], by mechanisms as yet undefined. Significantly, we reported that bacopaside II blocked the aquaporin-1 (AQP1) water channel and impaired the in vitro migration of HT-29 colon cancer cells that express comparatively high levels of AQP1, at concentrations that were not appreciably cytotoxic, but had minimal effect on migration of SW480, which expressed lower levels [12]. The aim of this study was to further elucidate the potential anti-tumour effects of bacopaside II in colorectal cancer cells in vitro.

## 2. Materials and Methods

### 2.1. Cell Lines and Culture

HT-29, SW480, SW620 and HCT116 colon cancer cells were obtained from American Type Culture Collection (ATCC, Manassas, VA, USA) and maintained in culture medium consisting of DMEM (Life Technologies, Eugene, OR, USA) supplemented with 10% heat-inactivated foetal bovine serum (FBS; Sigma-Aldrich, St. Louis, MO, USA), 200 U/mL penicillin, 200 µg/mL streptomycin (Life Technologies) and 2 mM l-alanyl-l-glutamine dipeptide (GlutaMAX Supplement; Life Technologies), and incubated at 37 °C with 5% CO_2_ in air. All cells were mycoplasma-free (MycoAlert mycoplasma detection kit; Lonza, Basel, Switzerland).

### 2.2. Analysis of AQP1 Expression by Quantitative PCR and by Western Immunoblot

Cells were seeded at 5 × 10^5^ cells per well in six well plates and incubated for 24 h before isolation of either total RNA or protein. Total RNA was isolated using the DNA/RNA/miRNA Universal Kit with DNase I on-column digestion (Qiagen, Hilden, Germany). Total RNA (1 µg) was reverse transcribed using the iScript cDNA Synthesis Kit (Bio-Rad Laboratories, Hercules, CA, USA) in a final volume of 20 µL. Transcript expression was determined using multiplex TaqMan Gene Expression Assays for AQP1 (Hs01028916_m1) and phosphomannose mutase 1 (PMM1; Hs00963625_m1; Applied Biosystems, Foster City, CA, USA). Reactions were performed using a CFX96 Thermal Cycler (Bio-Rad) with activation for 30 s at 95 °C followed by 40 cycles of 15 s at 95 °C and 30 s at 60 °C. Each 20 μL reaction consisted of 10 μL of SsoAdvanced Universal Probes Supermix (Bio-Rad), 1 μL of each 20 x TaqMan Gene Expression Assay, and 1 μL of cDNA. Results were calculated using the ΔΔCt relative quantification method, normalising to PMM1 reference gene.

Immunoblotting was performed essentially as previously described [14,15]. Cells were lysed with RIPA Lysis and Extraction Buffer (Thermo Fisher Scientific, Waltham, MA, USA) supplemented with Halt Protease Inhibitor Cocktail (Thermo Fisher Scientific) on ice for 10 min, homogenised by passing through a 26-gauge needle, and centrifuged at 17,000× *g* for 15 min at 4 °C to pellet cell debris. Protein was quantified using the Bio-Rad Protein Assay (Bio-Rad). Protein (50 μg) was resolved by denaturing electrophoresis using 12% Mini-PROTEAN TGX Stain-Free precast gels and transferred to 0.2 µm polyvinylidene difluoride membranes using the Trans-Blot Turbo Transfer System (Bio-Rad). Membranes were blocked with tris-buffered saline (TBST; 20 mM Tris, 500 mM NaCl, 0.05% tween 20) supplemented with 4% (*w*/*v*) skim milk powder for 1 h at room temperature, then immunostained by incubating overnight at 4 °C with 1:1000 rabbit monoclonal antibody to AQP1 (EPR11588(B); Abcam, Cambridge, UK), followed by 1 h at room temperature with 1:2000 goat anti-rabbit IgG horse radish peroxidase (HRP) secondary antibody and 1:10,000 streptactin-HRP conjugate (Bio-Rad) in TBST supplemented with 1% (*w*/*v*) skim milk powder at room temperature for 1 h. Membranes were then immunostained for 1 h at room temperature with 1:5000 mouse polyclonal antibody to beta-actin (clone AC-15; Sigma-Aldrich) followed by 1:2000 goat anti-mouse IgG HRP (Bio-Rad) [16]. Clarity Western ECL Blotting Substrate was applied and membranes imaged using a ChemiDocTouch Imaging System (Bio-Rad). Image Lab v6.0 (Bio-Rad) was used to quantify the bands, normalising to either beta-actin or total protein loading [17].

### 2.3. Cell Growth Assay

Cell growth was determined by crystal violet assay as described previously [18]. Briefly, 10^3^ cells per well were seeded into 96-well plates and cultured for 24 h. Culture medium without cells was added to 6 wells to serve as background controls for nonspecific binding of the crystal violet dye. Cells were treated for 72 h with either 0, 2.5, 5, 10, 15, 20 or 30 µM of bacopaside II analytical standard (Sigma-Aldrich) dissolved in 2% (*v*/*v*) methanol vehicle. Cells were fixed with 10% neutral buffered formalin for 30 min, stained with 1% (*w*/*v*) crystal violet (Sigma-Aldrich) in 2% ethanol for 10 min, washed eight times in running distilled water, and air-dried. Crystal violet was eluted using 10% acetic acid with gentle rocking of the plates for 1 h at room temperature. Absorbance of the eluent was measured at 595 nm using a FLUOstar Optima microplate reader (BMG Labtech, Ortenberg, Germany). The average absorbance of the wells without cells was subtracted from the absorbance of each of the wells containing cells, and the data were expressed as the mean absorbance relative to that of the vehicle control treated cells.

### 2.4. Acridine Orange Staining

Cells were seeded at 5 × 10^5^ cells per well in six-well plates and incubated for 24 h. Cells were washed with Dulbecco’s phosphate buffered saline (DPBS; Life Technologies) to remove non-viable cells, and then treated for 24 h with either 0, 5, 10, 15, 20 or 30 µM of bacopaside II analytical standard (Sigma-Aldrich) dissolved in 2% (*v*/*v*) methanol vehicle. Non-adherent and adherent cells were harvested and pooled. To harvest adherent cells, the cells were washed three times with DPBS, collecting each wash, and then incubated with 0.25% trypsin-EDTA (Life Technologies) at 37 °C until the cells had detached. The trypsin-EDTA was inactivated with culture medium supplemented with 10% FBS. Cells were washed twice with DPBS by centrifuging at 200× *g* for 10 min at 4 °C and aspirating the supernatant. Cells were stained with 1 μg/mL acridine orange (Sigma-Aldrich) in DPBS at 37 °C for 15 min and immediately analysed using a FACSCanto II (BD Biosciences, San Jose, CA, USA) flow cytometer, acquiring at least 50,000 single cell events per sample.

### 2.5. Cell Cycle Analysis by Propidium Iodide Staining

Cells were seeded at 5 × 10^5^ cells per well in six-well plates, treated with bacopaside II, and harvested as described above. Cells were washed twice with DPBS and resuspended in 1.2 mL of ice cold DPBS in polypropylene flow cytometry tubes. Next, 2.8 mL of 100% ice cold ethanol was added dropwise with gentle vortexing, to achieve a final concentration of 70% ethanol. The fixed cells were stored at −20 °C overnight, washed twice by centrifuging at 200× *g* for 10 min at 4 °C and aspirating the supernatant. Cells were resuspended in freshly prepared propidium iodide (PI) staining solution consisting of 200 µg/mL PI (Sigma-Aldrich), 200 µg/mL DNase-free RNase A (Sigma-Aldrich), and 0.1% (*v/v*) triton X-100 (Sigma-Aldrich) in DPBS, incubated at 37 °C for 15 min, and then placed on ice protected from light. Stained cells were analysed using a FACSCanto II (BD Biosciences) flow cytometer, acquiring at least 50,000 single cell events per sample. Quantification of the percentage of cells in G0/G1, S, and G2/M phases of the cell cycle was performed using the Watson (Pragmatic) model in FlowJo v10.4.1 (FlowJo, LLC, Ashland, OR, USA).

### 2.6. Apoptosis Assay by Annexin V Propidium Iodide Staining 

Cells were seeded at 5 × 10^5^ cells per well in six-well plates, treated with bacopaside II, and harvested as described above. Cells were washed twice with DPBS and stained with the Annexin-V-FLUOS staining kit (Roche Diagnostics, Mannheim, Germany) following the manufacturer’s instructions. To compensate for the overlapping spectra of annexin V and PI, additional unlabelled and single-labelled samples, which contained dead cells, were prepared. Necrotic cells were prepared by heating a cell suspension in DPBS at 63 °C for 30 min. Cells were analysed using a FACSCanto II (BD Biosciences), gating out debris and doublets, and acquiring at least 10,000 single cell events per sample. Quantification of viable (double-negative), early apoptotic (annexin V-positive), late apoptotic (annexin V and PI double-positive) and necrotic cells (PI-positive) was performed using FlowJo v10.4.1 (FlowJo, LLC). 

### 2.7. Statistical Analysis

Statistical analysis and the half maximal inhibitory concentrations (IC50) were determined using Prism version 7.0d for Mac OS X (GraphPad Software, La Jolla, CA, USA). An ordinary one-way analysis of variance (ANOVA) with Tukey correction for multiple comparisons was used to analyse the quantitative PCR and western immunoblot data, comparing all cell lines to each other. For the cell growth data, an ordinary one-way (ANOVA) with Dunnett’s correction for multiple comparison was used to compare each concentration to 0 µM bacopaside II (vehicle alone).

## 3. Results

### 3.1. Expression of Aquaporin-1 in Colon Cancer Cell Lines

**Figure 1:**
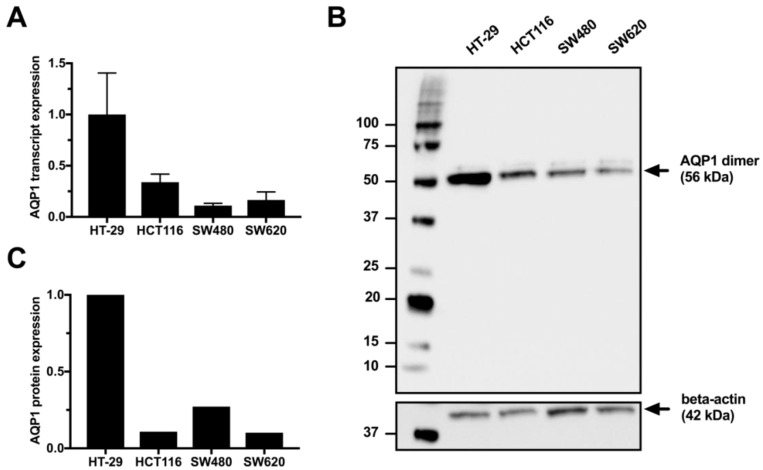
(**A**) Relative AQP1 transcript expression in untreated HT-29, HCT116, SW480, and SW620 colon cancer cell lines. Transcript expression was calculated using the ΔΔCt relative quantification method, normalising to PMM1 reference gene. Results are the mean ± SD of biological triplicates, relative to the expression in HT-29. (**B**) Representative western immunoblot for AQP1 protein expression in untreated HT-29, HCT116, SW480, and SW620 colon cancer cell lines. (**C**) Relative AQP1 protein expression in untreated HT-29, HCT116, SW480, and SW620 colon cancer cell lines. AQP1 expression was normalised to beta-actin loading control and relative to the expression in HT-29.

Expression of AQP1 was determined by quantitative PCR and western immunoblot in the untreated colon cancer cell lines HT-29, HCT116, SW480, and SW620 (Figure 1 and Supplementary Figure S1). Transcript expression of AQP1 was significantly higher in HT-29 compared to either HCT116 (*p* = 0.0207), SW480 (*p* = 0.0038) or SW620 (*p* = 0.0056) (Figure 1A). Western immunoblots demonstrated that unlike red blood cells (RBC) which had both monomeric (28 kDa) and glycosylated (30–40 kDa) forms (Supplementary Figure S2), the predominant form observed in colon cancer cell lines was the 56 kDa dimer, consistent with previous reports describing AQP1 in RBC, HT-29, SW480 and HCT116 [12,15,19]. Protein expression of AQP1 was higher in HT-29 compared to either HCT116, SW480, or SW620, when AQP1 expression was normalised to beta-actin (Figure 1C) or total protein loaded (Supplementary Figure S1). There were no significant differences in AQP1 expression between SW480, SW620 and HCT116.

### 3.2. Bacopaside II Inhibited Colon Cancer Cell Growth

**Figure 2:**
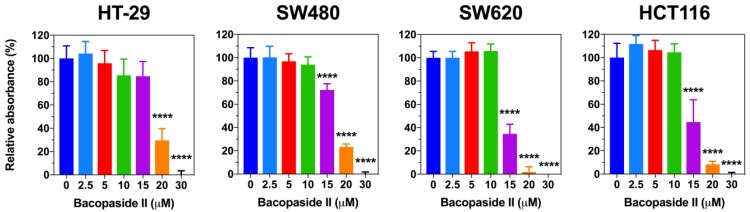
Effect of bacopaside II on adherent colon cancer cell growth. Cells were treated with bacopaside II for 72 h and then cell numbers were determined using crystal violet staining. Data are the mean ± SD of six replicates, relative to vehicle control (0 µM bacopaside II) treated cells. **** *p* < 0.0001.

To determine the effect of bacopaside II on colon cancer cell growth, each cell line was treated with various doses of bacopaside II for 72 h and then stained with crystal violet (Figure 2). Significant reductions in adherent cell growth were observed after treatment with bacopaside II at concentrations of ≥15 µM for SW480, SW620 and HCT116 and ≥20 µM for HT-29. The estimated cell growth IC50 for HT-29, SW480, SW620 and HCT116 was 18.4, 17.3, 14.6 and 14.5 µM respectively. Quantitative PCR and western immunoblot were performed on each cell line following 24 h of treatment with either vehicle or 15 µM bacopaside II. A significant increase in AQP1 transcript and protein expression was observed in HT-29, but not in HCT116, SW480 or SW620 (Supplementary Figure S3).

### 3.3. Bacopaside II Induced Morphological Changes

**Figure 3:**
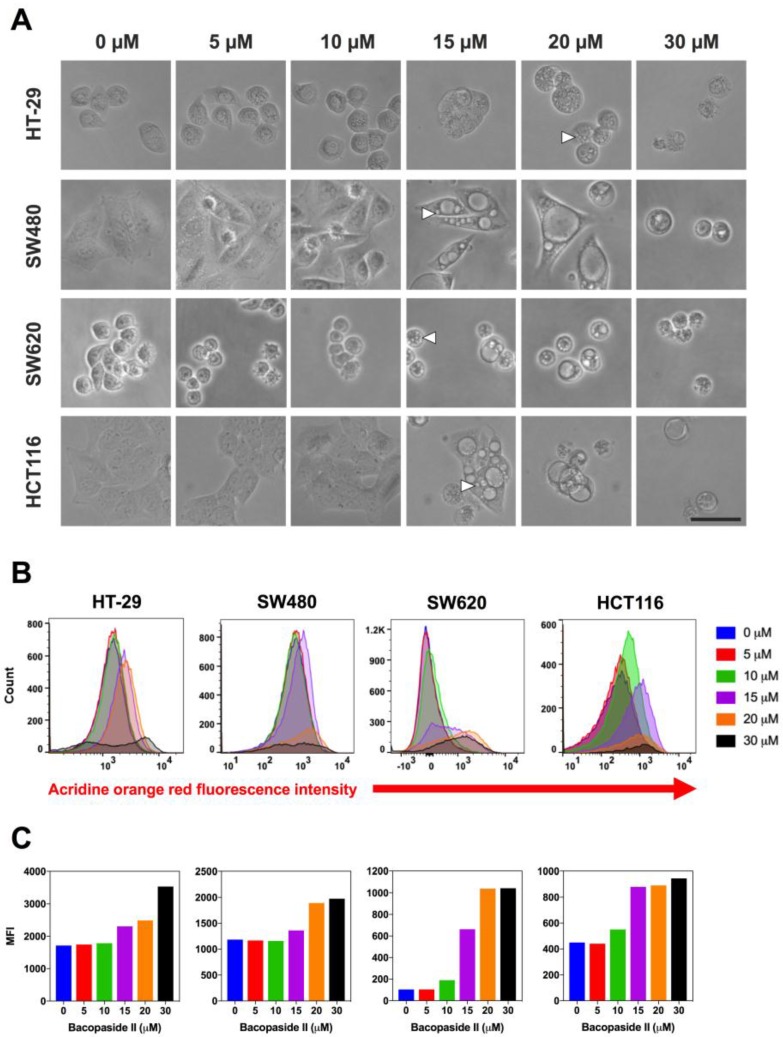
(**A**) Effect of bacopaside II on colon cancer cell morphology. Cells were treated for 48 h with various doses of bacopaside II. Intracellular vacuoles (white arrows) were prominent in HT-29 at 20 μM and in SW480, SW620 and HCT116 at 15 μM. Magnification 200 x, scale bar = 25 μm. (**B**) Effect of bacopaside II on acridine orange staining. Cells were treated with bacopaside II for 24 h, stained with acridine orange, and red fluorescence intensity was analysed by flow cytometry showing representative histograms of the red fluorescence intensity. (**C**) Red mean fluorescence intensity (MFI) values derived from the histograms.

To elucidate the potential mechanisms of bacopaside II induced inhibition of colon cancer cell growth, we monitored morphological changes using time-lapsed microscopy (Figure 3). Treatment with bacopaside II resulted in the appearance of prominent intracellular vacuoles as early as 6 h in SW480, SW620 and HCT116 and by 18 h in HT-29. The appearance of these vacuoles coincided with a noticeable inhibition of cell division. Intracellular vacuoles were evident at concentrations of bacopaside II that inhibited colon cancer cell growth, ≥20 µM for HT-29 and ≥15 µM for SW480, SW620 and HCT116. Bacopaside II at 30 µM caused a considerable percentage of cells to detach by 24 h for each of the colon cancer cell lines, together with notable cellular shrinkage and blebbing without lysis, consistent with apoptosis. The few remaining adherent cells had intracellular vacuoles.

### 3.4. Bacopaside II Increased Acridine Orange Staining

To investigate if the intracellular vacuoles induced by bacopaside II were autophagic vacuoles, cells were treated with bacopaside II for 24 h, stained with acridine orange, and then analysed by flow cytometry (Figure 3B,C). In acridine orange-stained cells, the cytoplasm and nucleolus fluoresce bright green and dim red, whereas acidic compartments fluoresce bright red. The intensity of the red fluorescence is proportional to the degree of acidity and/or the volume of the cellular acidic compartment [20]. Bacopaside II increased the red mean fluorescence intensity (MFI) in HT-29 and SW480 at ≥15 μM and in SW620 and HCT116 at ≥10 μM. Together with the observed morphological changes, these results suggest that bacopaside II may have induced autophagy in these colon cancer cells.

### 3.5. Bacopaside II Induced Cell Cycle Arrest

**Figure 4:**
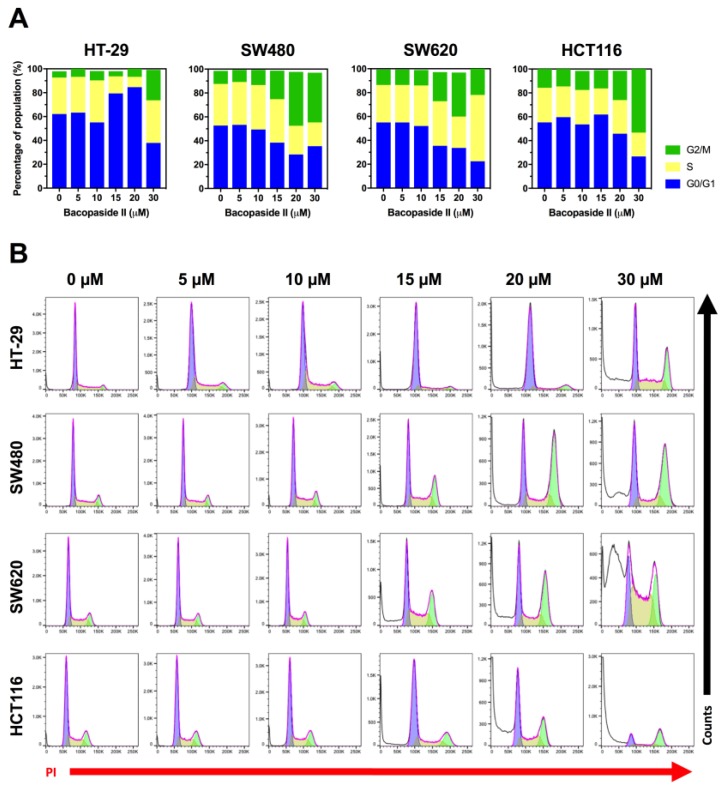
Effect of bacopaside II on cell cycling. Cells were treated with bacopaside II for 24 h, the DNA was stained with propidium iodide, and analysed by flow cytometry. (**A**) The percentage of population of single cells in G0/G1, S and G2/M phases of the cell cycle. (**B**) Representative histograms of the PI stained DNA, showing the subG1 events (white), G0/G1 (blue), S (yellow) and G2/M (green). Results shown are representative of 3 repeated experiments.

Having observed inhibition of cell growth, we investigated if bacopaside II induced cell cycle arrest using flow cytometric analysis of propidium iodide stained DNA (Figure 4). Treatment with bacopaside II at ≥15 μM for SW480 and SW620, and at ≥20 μM for HCT116 resulted in an increase in the percentage of cells in G2/M and a concomitant decrease in G0/G1. In contrast, for HT-29, bacopaside II at 15 and 20 μM caused an increase in G0/G1 and a decrease in S, whilst 30 μM caused an increase in G2/M and a concomitant decrease in G0/G1 (Figure 4A). Treatment with bacopaside II at ≥20 μM for HT-29, and at ≥15 μM for SW480, SW620 and HCT116 induced a marked increase in the number of subG1 events, suggestive of cell death and consistent with the observed reductions in cell growth (Figure 4B).

### 3.6. Bacopaside II Induced Apoptosis

**Figure 5:**
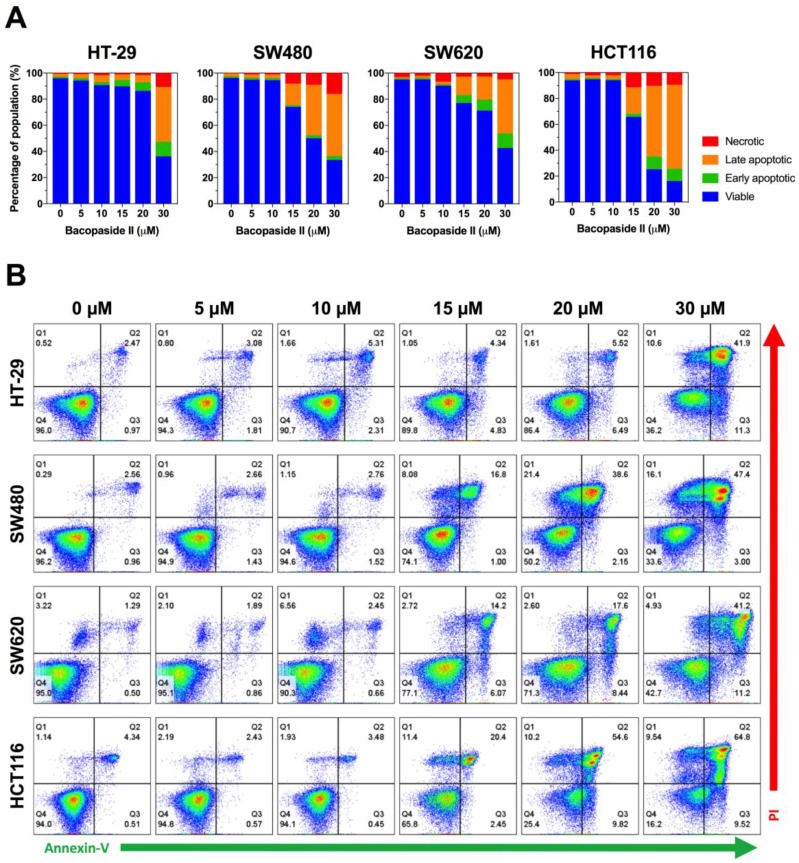
Effect of bacopaside on apoptosis. Cells were treated with bacopaside II for 24 h, stained with the Annexin-V-FLUOS staining kit, and analysed by flow cytometry. (**A**) Representative plots, showing viable (Q4, double negative), early apoptotic (Q3, annexin V positive), late apoptotic (Q2, annexin V and PI positive) and necrotic cells (Q1, PI positive). (**B**) Percentage of the population of single cells in each quadrant. Results shown are representative of 3 repeated experiments.

To determine if bacopaside II inhibited cell growth by inducing apoptosis, cells were stained using the Annexin-V-FLUOS staining kit. Exposure of phosphatidylserine to the outer leaflet of the plasma membrane occurs early in apoptosis and can be detected by flow cytometry using a fluorescently conjugated annexin V, which binds to phosphatidylserine. Viable cells do not stain (annexin V and PI double-negative), early apoptotic cells display increased staining only with annexin V (annexin V positive, PI negative), for late apoptotic cells the cell membrane integrity is lost allowing penetration of PI (annexin V and PI double-positive), and necrotic cells stain with PI only (annexin V negative, PI positive). Bacopaside II induced an increase in annexin V staining in each of the colon cancer cell lines, indicative of apoptosis (Figure 5). For HT-29 cells, 30 μM bacopaside II induced a marked increase in the percentage of early and late apoptotic cells; in contrast, for SW480, SW620 and HCT116 cells this marked increase occurred with ≥15 μM bacopaside II.

## 4. Discussion

This novel study investigated the anti-tumour effects of the AQP1 water channel inhibitor bacopaside II on colon cancer cell lines in vitro. We assessed its effects on cell growth, morphology, acridine orange staining, cell cycling and apoptosis in HT-29, SW480, SW620 and HCT116 colon cancer cell lines. Bacopaside II inhibited cell growth by inducing cell cycle arrest and apoptosis. The distinctive morphological change and increase in acridine orange staining suggested that bacopaside II might also have induced autophagy. Expression of AQP1 varied between the cell lines, comparatively high in HT-29 and low in SW480, SW620 and HCT116. The responses to bacopaside II also varied between the cell lines. Bacopaside II significantly inhibited the cell growth of HT-29 at ≥20 µM and of SW480, SW620 and HCT116 at ≥15 µM. Growth inhibition of HT-29 at 20 µM was primarily mediated by a G0/G1 cell cycle arrest, and at 30 µM by a G2/M cell cycle arrest and apoptosis. In contrast in SW480, SW620 and HCT116, growth inhibition at ≥15 µM was mediated by a G2/M cell cycle arrest and apoptosis. Together, these results provide further evidence that the observed effects of bacopaside II might be dependent on the levels of AQP1 expression in colon cancer cells.

We reported previously, using a combination of quantitative oocyte swelling, two-electrode voltage clamp techniques, and in silico molecular modelling, that bacopaside II selectively blocked the AQP1 water channel by occluding the cytoplasmic side of the water pore without impairing ionic conductance [12]. Furthermore, bacopaside II impaired migration of the high AQP1 expressing HT-29 but had minimal effect on the low expressing SW480, raising the possibility that the observed effects on migration were dependent on the levels of AQP1 expression. However, at this point, we cannot rule out that the effects on cell growth and migration are mediated by other targets, either independently or in conjunction with AQP1. For example, bacopaside II inhibits the activity of P-glycoprotein, an ATP-dependent efflux pump involved in transporting a broad range of xenobiotics across extra- and intracellular membranes [21]. Furthermore, HT-29 harbors an activating BRAF mutation (BRAF^V600E^) and developed from a serrated polyp, in contrast to the remaining three cell lines. Whether these and other differences contribute to the observed differential effects of bacopaside II is currently unknown and requires further investigation. 

Previous studies have shown that bacopaside II is cytotoxic to cancer cells in vitro using assays that assessed mitochondrial metabolic activity, by mechanisms unknown. Consistent with the apoptosis results reported here, we previously reported that bacopaside II was cytotoxic to HT-29 cells after 24 h of treatment with 30 μM bacopaside II, using a resazurin-based alamarBlue assay [12]. Peng et al. demonstrated that bacopaside II was cytotoxic, as determined by methyl thiazole tetrazolium assay in human breast, brain, ileocecal, lung and prostate cancer cells with IC50s of 32–45 μM depending on the cell line [13]. However, they reported that concentrations of bacopaside II either side of the cytotoxic IC50 (25 and 50 μM) failed to significantly reduce cell adhesion, matrigel invasion, or migration of breast cancer cells in vitro. The reasons for this disparity are unknown.

We report for the first time that bacopaside II induced morphological changes and increased acridine orange red mean fluorescence intensity in colon cancer cells, findings that are consistent with the induction of autophagy. Interestingly, the increase in acridine orange staining was detected at or below the lowest concentration capable of inducing either cell cycle arrest or apoptosis. Whilst alcoholic extracts of *Bacopa monnieri* have been shown to protect against benzo[a]pyrene induced apoptosis in immortalised human keratinocytes through beclin-1-dependant autophagy activation [22], this is the first study to suggest that bacopaside II might induce autophagy.

## 5. Conclusions

In conclusion, we demonstrate for the first time that bacopaside II inhibits colon cancer cell growth in vitro by inducing cell cycle arrest and apoptosis. The differences in the dose response and AQP1 expression between the different colon cancer cell lines raises the possibility that the observed effects are influenced by the levels of AQP1 expression. Together with our previously reported findings that bacopaside II inhibits migration of colon cancer cells, these findings suggest that bacopaside II might have promising anti-cancer activity for the treatment of colorectal and other cancers.

## Supplementary Materials

The following are available online at http://www.mdpi.com/2073-4409/7/7/81/s1.
