# Supplementary material for: The Purified Extract from the Medicinal Plant Bacopa monnieri, Bacopaside II, Inhibits Growth of Colon Cancer Cells In Vitro by Inducing Cell Cycle Arrest and Apoptosis
Source: Cells. 2018 Jul 21;7(7):81. doi: 10.3390/cells7070081 (PMC6070819; doi:10.3390/cells7070081)
Supplement: Supplementary file 1 [file cells-07-00081-s001.pdf]

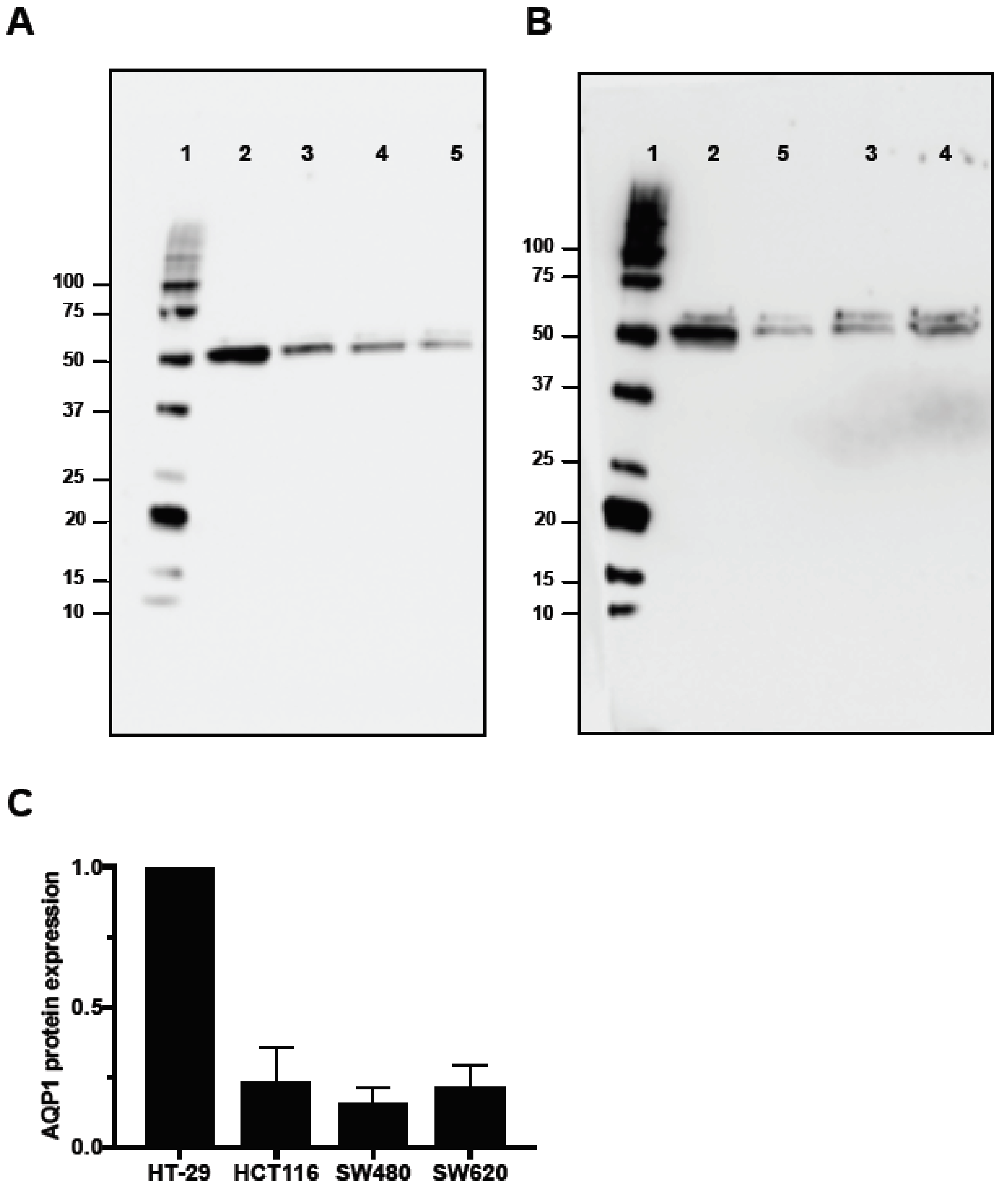

Supplementary Figure 1: Western immunoblots for AQP1 expression in colon cancer cell lines. On two separate occasions (A and B) the cells were seeded into 6-well plates and the western immunoblots were performed as described in the Materials and Methods. 1: molecular weight marker; 2: HT-29; 3: HCT116; 4: SW480; 5: SW620. (C) AQP1 protein expression was determined by normalizing to total protein loading and relative to expression in HT-29. Results are the mean +/- SD for the two independent westerns (A and B). AQP1 protein expression was significantly higher in HT-29 compared to either HCT116 ($p = 0.0021$), SW480 ($p = 0.0015$), or SW620 ($p = 0.002$).

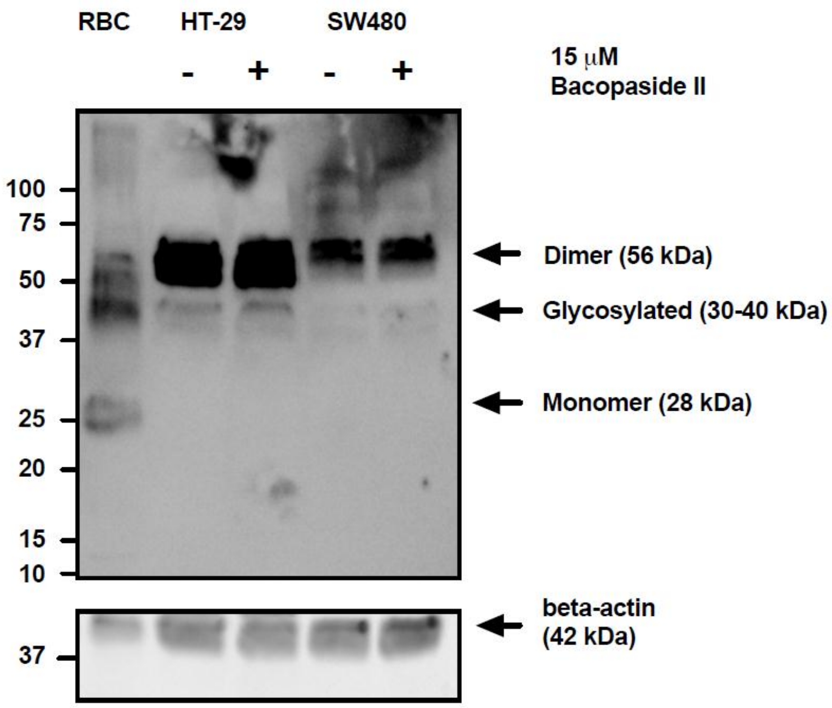

Supplementary Figure 2: Western immunoblot for AQP1 expression red blood cells and HT-29 and SW480. Total protein was resolved using SDS-PAGE on 12% Mini-PROTEAN TGX Stain-Free precast and immunostained for AQP1 and beta-actin as described in the Materials and Methods.

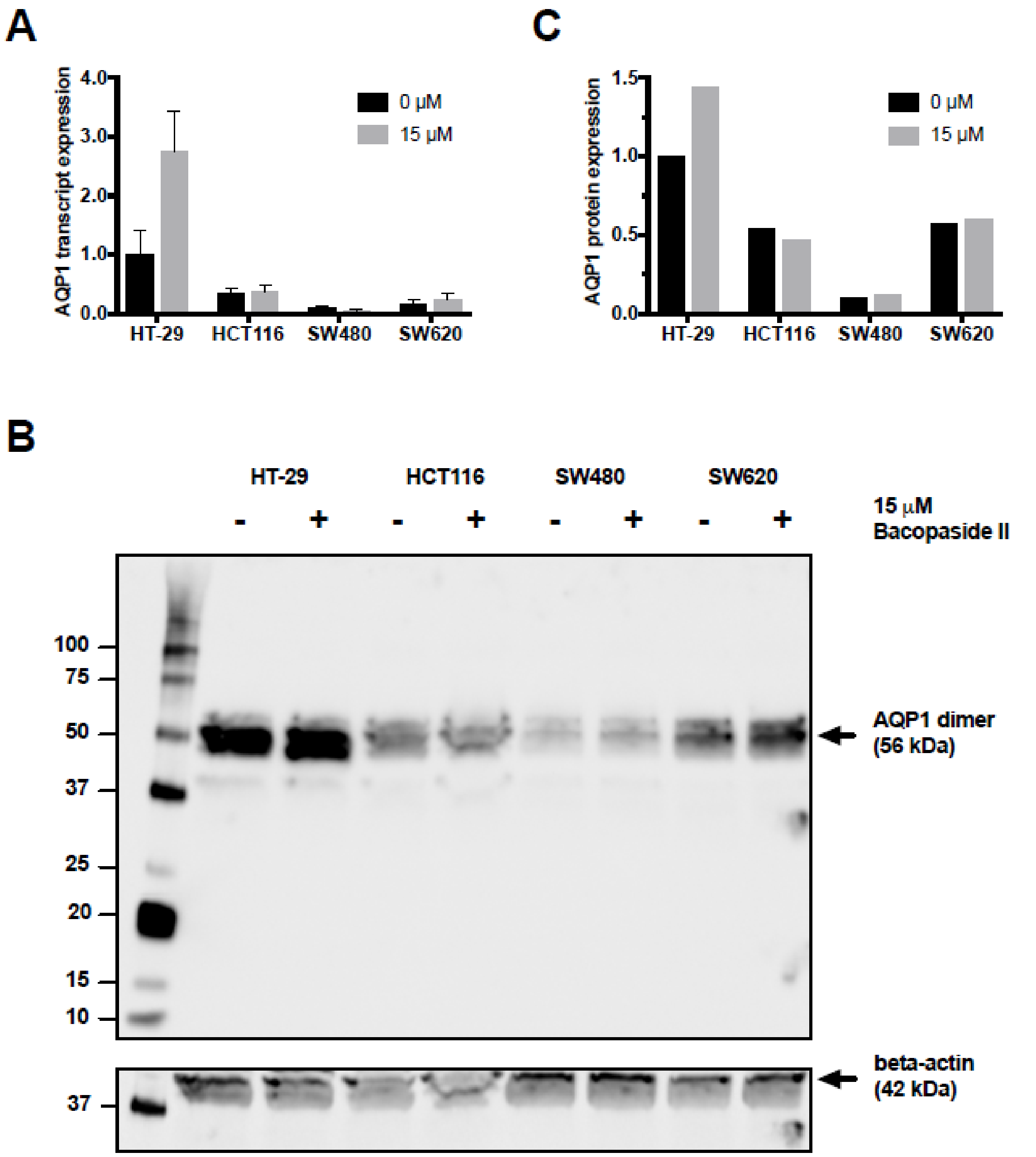

Supplementary Figure 3: AQP1 expression in colon cancer cell lines treated with bacopaside II. Cells were treated with either vehicle (0 µM) or bacopaside II (15 µM) for 24 h and then either total RNA or protein was harvested. (A) AQP1 transcript expression normalised to the reference gene PMM1 and relative to HT-29. (B) Representative western immunoblot for AQP1 and beta-actin. (C) AQP1 protein expression normalised to beta-actin and relative to HT-29.
